# Acute Sheehan Syndrome Presenting as Panhypopituitarism and Reversible Cardiac Dysfunction in a Patient With Sickle Cell Disease: A Case Report

**DOI:** 10.7759/cureus.117047

**Published:** 2026-09-28

**Authors:** Rawan Abdulrahman, Alya Alhajjaj

**Affiliations:** 1 Internal Medicine, Mouwasat Hospital, Dammam, SAU; 2 Internal Medicine, Royal College of Surgeons in Ireland, Busaiteen, BHR; 3 Internal Medicine and Endocrinology, Qatif Central Hospital, Qatif, SAU

**Keywords:** cardiomyopathy, panhypopituitarism, postpartum hemorrhage, sheehan syndrome, sickle cell disease

## Abstract

Sheehan syndrome is an uncommon cause of hypopituitarism resulting from ischemic pituitary necrosis following severe postpartum hemorrhage (PPH) and hypotension. Its presentation can be delayed and non-specific, and it is rarely reported in the context of mild PPH with severe hemolysis. Here, we describe a 31-year-old woman with underlying sickle cell disease (SCD) who developed panhypopituitarism and reversible cardiac dysfunction after an 11-week period of progressive symptoms following spontaneous vaginal delivery complicated by an estimated blood loss of 700 mL and severe hemolysis. Pituitary hormonal replacement therapy, together with heart failure treatment, resulted in marked clinical and biochemical improvement, with normalization of metabolic abnormalities and recovery of cardiac function. This case suggests a possible association between Sheehan syndrome and postpartum blood loss of 700 mL accompanied by severe hemolysis, though this remains hypothetical based on a single case.

## Introduction

Sheehan syndrome is a form of hypopituitarism caused by ischemic necrosis of the pituitary gland, classically occurring after severe postpartum hemorrhage (PPH) and hypotension [1]. The underlying pathophysiology is related to pituitary enlargement during pregnancy and the relative vulnerability of its low-pressure portal blood supply, predisposing the gland to ischemic injury in the setting of severe hemodynamic compromise [1]. Acute Sheehan syndrome refers to the onset of clinical and biochemical hypopituitarism within approximately six weeks of delivery [2].

The American College of Obstetricians and Gynecologists defines PPH as a cumulative blood loss ≥1000 mL within 24 hours of birth or any blood loss associated with signs of hypovolemia [3]. While the disorder is traditionally associated with substantial obstetric blood loss, hypopituitarism has been reported in some cases without clearly quantified or massive hemorrhage [4]. It has further been hypothesized that severe hemolysis and anemia may represent an additional contributor to pituitary ischemia in the absence of massive hemorrhage [5], though this mechanism remains unproven. To our knowledge, this case represents a rare presentation of acute Sheehan syndrome occurring with a documented blood loss below conventional PPH thresholds, in a patient with SCD-associated severe hemolysis; a combination that has not been previously well characterized in the literature.

Clinical presentation is often insidious and non-specific, which frequently delays diagnosis by weeks to years. Cardiac involvement, in the form of cardiac dysfunction, is a rare but recognized manifestation of severe hypopituitarism and has been associated with deficiencies in growth hormone and thyroid hormone. Cardiac dysfunction may improve following appropriate hormone replacement.

We report a case of acute Sheehan syndrome in a patient with sickle cell disease (SCD) presenting with panhypopituitarism and reversible cardiac dysfunction following a delivery complicated by an estimated blood loss of 700 mL and severe hemolysis. This case highlights the possibility that severe hemolysis and SCD-related microvascular pathology may contribute to pituitary ischemia even in the absence of massive PPH.

## Case presentation

A 31-year-old Saudi female, gravida 4 para 3, with SCD, delivered at 38 weeks' gestation via spontaneous vaginal delivery, presented with an 11-week history of progressive nausea, vomiting, dizziness, and palpitations, with an estimated blood loss of 700 mL. Her pregnancy was reportedly uncomplicated, with no documented gestational diabetes, hypertensive disorders, or SCD-related crises; detailed antenatal and intrapartum vital signs were not available for review. Her pre-delivery baseline hemoglobin was 9 g/dL. Retained placental tissue was suspected at the time but excluded on subsequent evaluation. She denied visual disturbance or relevant medication use around the time of delivery, and reported no cardiac symptoms, such as dyspnea, orthopnea, or palpitations, prior to delivery. On postpartum day 1, she developed headache, dyspnea, dizziness, and palpitations attributed to anemia (hemoglobin 6.6 g/dL) and received a blood transfusion; her hemoglobin decline was attributed to hemolysis rather than significant obstetric blood loss, consistent with an underlying SCD-related hemolytic process. There was no documented hypotension, syncope, or shock requiring intensive-level care, and her vital signs prior to discharge were stable (BP 150/87 mmHg, pulse 84/min, no postural changes). Laboratory findings suggested hemolysis, and she was discharged after transient improvement.

Over the subsequent weeks, her symptoms persisted and progressively worsened, with the subsequent development of fatigue, cold intolerance, dry skin, failure to lactate, and amenorrhea. She was admitted on two further occasions to other facilities for anemia and received multiple additional blood transfusions; detailed vital signs from these admissions were not available for review. She was also admitted to our institution once prior to the index presentation, for severe anemia, during which she was noted to be tachycardic with otherwise stable vital signs, and was again transfused and discharged.

On presentation, she was tachycardic with orthostatic hypotension (BP 105/69 mmHg supine and 90/70 mmHg standing; pulse 133 beats/min). She was pale and jaundiced, with dry skin and cold extremities, without dehydration or hyperpigmentation. The remainder of the examination was unremarkable.

Investigations revealed severe anemia with evidence of ongoing hemolysis, marked hyponatremia, and hypoglycemia (Table 1). Serum sodium was markedly reduced at 117 mEq/L compared with her baseline sodium of 141 mEq/L. Hormonal evaluation demonstrated central hypothyroidism, secondary adrenal insufficiency, hypogonadotropic hypogonadism, and an inappropriately low prolactin level for the postpartum state.

**Table 1:** Laboratory investigations on presentation

Laboratory parameter	Patient value	Reference range
Hemoglobin	5.2 g/dL	12-15 g/dL
Mean corpuscular volume (MCV)	74 fL	80-100 fL
Red cell distribution width (RDW)	21%	11.5%-14.5%
Reticulocyte count	21.4%	0.5%-2.5%
Total bilirubin	133 µmol/L	2-20 µmol/L
Lactate dehydrogenase (LDH)	799 U/L	140-280 U/L
Aspartate aminotransferase (AST)	176 U/L	5-30 U/L
Ferritin	339 ng/mL	12-150 ng/mL
Transferrin saturation	39%	20%-50%
Sodium	117 mEq/L	135-145 mEq/L
Potassium	3.5 mEq/L	3.5-5.0 mEq/L
Random blood glucose	45 mg/dL	65-110 mg/dL
Free thyroxine (FT4)	0.35 ng/dL	0.8-1.8 ng/dL
Free triiodothyronine (FT3)	2.82 pg/mL	1.7-3.7 pg/mL
Thyroid-stimulating hormone (TSH)	0.45 mIU/L	0.4-4.0 mIU/L
Adrenocorticotropic hormone (ACTH)	0.22 pmol/L	1.6-13.9 pmol/L
Morning cortisol	5.1 nmol/L	138-690 nmol/L
Luteinizing hormone (LH)	2.0 mIU/mL	2-12 mIU/mL
Follicle-stimulating hormone (FSH)	3.6 mIU/mL	3-10 mIU/mL
Estradiol	16 pg/mL	30-400 pg/mL
Prolactin	6.5 ng/mL	5-25 ng/mL

Collectively, these findings supported a diagnosis of panhypopituitarism.

Echocardiography demonstrated a reduced ejection fraction of 45%, global hypokinesia, mild pulmonary hypertension, and mild tricuspid regurgitation, raising the differential of hypopituitarism-related cardiac dysfunction versus peripartum or other cardiomyopathy. Given the postpartum history in combination with the clinical and biochemical features of pituitary dysfunction, an endocrine etiology was considered. Pituitary magnetic resonance imaging (MRI) was performed at this stage and demonstrated a small pituitary gland measuring approximately 4 mm in height, with no focal lesion, hemorrhage, or significant enhancement abnormality (Figure 1). The absence of a mass lesion or hemorrhage on MRI argued against a structural or infiltrative cause of hypopituitarism. The absence of pituitary enlargement or mass effect on initial MRI is more consistent with an ischemic process than with lymphocytic hypophysitis, which characteristically presents with gland enlargement in the acute phase. Although these findings were not characteristic of chronic Sheehan syndrome, the small pituitary gland, together with the clinical and biochemical findings, supported the diagnosis of acute postpartum hypopituitarism consistent with Sheehan syndrome.

**Figure 1:**
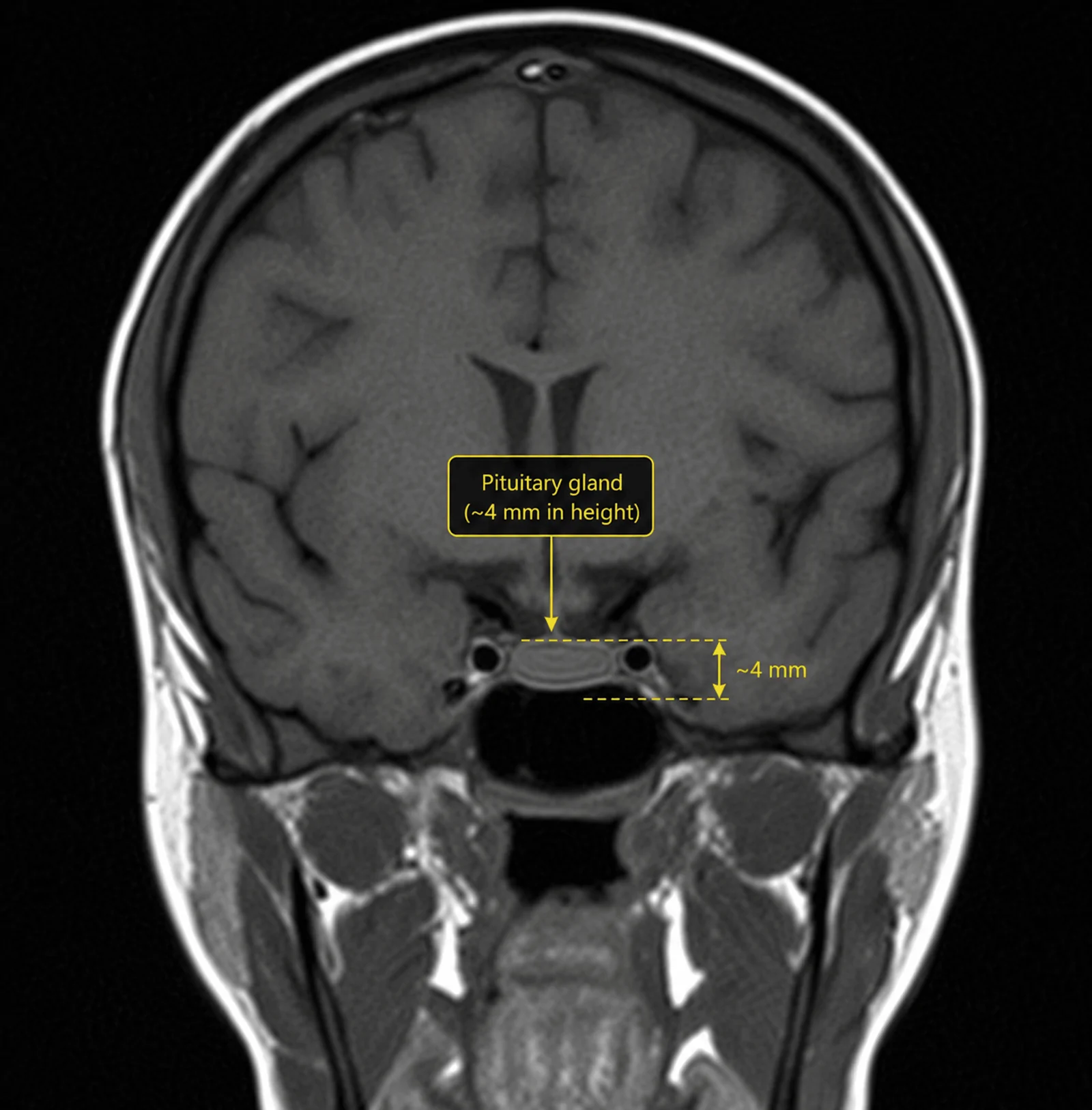
Brain MRI demonstrating a pituitary gland measuring approximately 4 mm in height, with no focal lesion, hemorrhage, or significant enhancement abnormality. No characteristic radiological features of chronic Sheehan syndrome were identified at this stage

She was diagnosed with panhypopituitarism secondary to acute Sheehan syndrome in the setting of SCD with severe hemolysis and an estimated postpartum blood loss of 700 mL. The reduced ejection fraction and global hypokinesia raised the possibility of cardiomyopathy secondary to hypopituitarism, particularly thyroid hormone deficiency, versus peripartum cardiomyopathy (PPCM). Because both conditions may occur in the postpartum period and may improve with treatment, the cardiac dysfunction was considered potentially multifactorial.

The acute presentation was managed with hemodynamic stabilization using intravenous fluids and blood transfusion. Hydrocortisone 100 mg was administered intravenously as an initial bolus, followed by 50 mg intravenously every six hours. Anti-heart failure medications, including bisoprolol, spironolactone, and telmisartan, were initiated. She was subsequently discharged on hydrocortisone 10 mg in the morning, 5 mg at noon, and 2.5 mg in the evening; levothyroxine 50 mcg daily; and anti-heart failure medications, with counseling on adrenal crisis emergency protocols and sick-day dose adjustments.

On follow-up, the patient showed significant clinical and biochemical improvement, with subsequent recovery of cardiac function following hormone replacement therapy. Hemoglobin stabilized at 9 g/dL. Thyroid function normalized on levothyroxine, and metabolic parameters, including renal and liver function, returned to normal.

Repeat echocardiography demonstrated normalization of ejection fraction and pulmonary pressures. She was commenced on combined estrogen-progestin replacement therapy. Fertility counselling was provided, including discussion of the potential need for ovulation induction in future pregnancies.

At one-year follow-up, pituitary MRI demonstrated further pituitary atrophy with an empty sella. This progressive change is consistent with, though not diagnostic of, pituitary infarction, as similar imaging evolution can occur in other causes of postpartum hypopituitarism, including lymphocytic hypophysitis (Figure 2).

**Figure 2:**
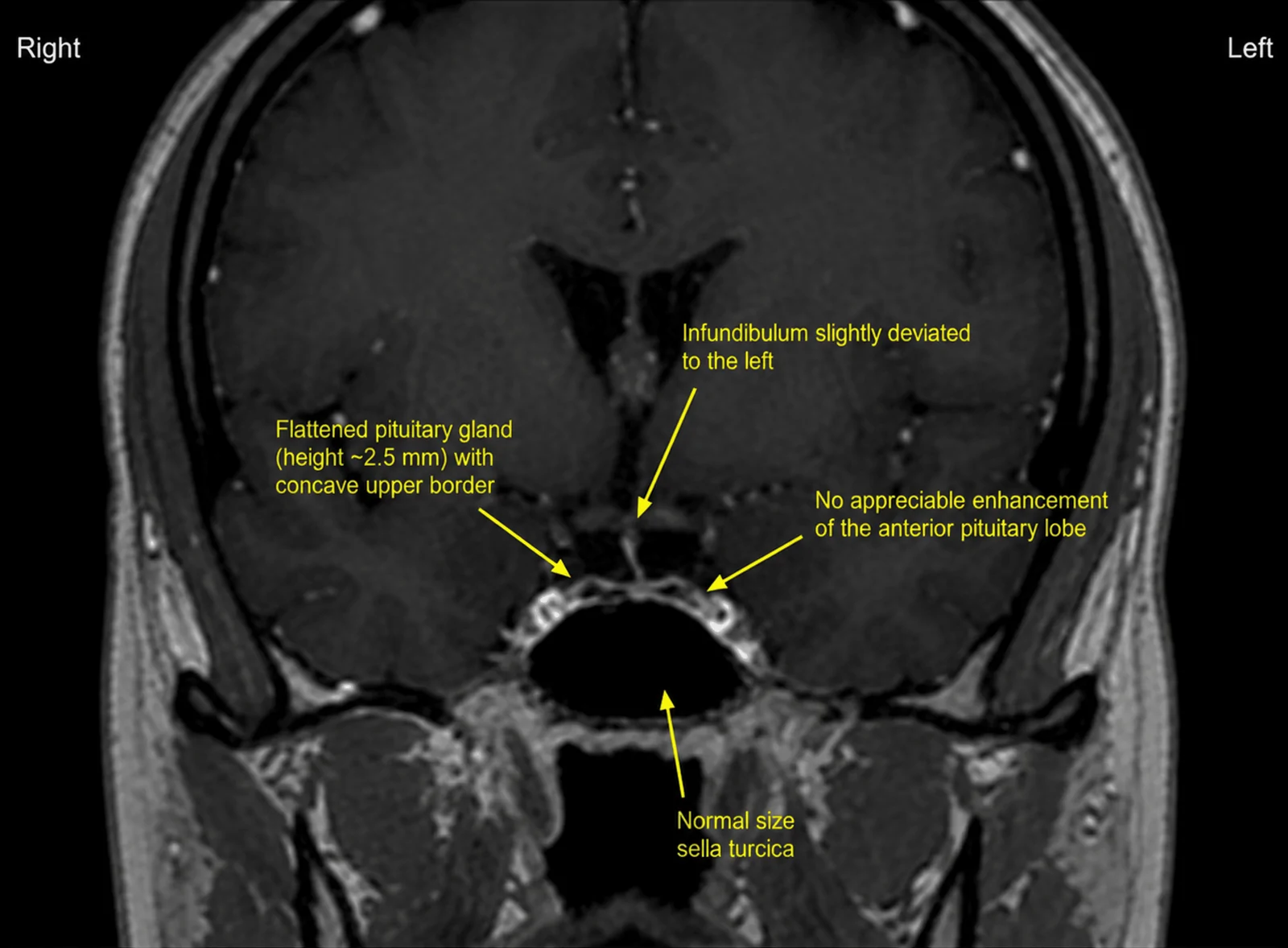
Brain MRI obtained one year after presentation demonstrating further pituitary atrophy, with the gland measuring approximately 2.5 mm in height and an appearance consistent with an empty sella

## Discussion

This case illustrates several important points. It demonstrates the diagnostic challenge of Sheehan syndrome in the context of SCD, its occurrence despite a documented postpartum blood loss below the conventional quantitative threshold for PPH (≥1000 mL) [3], and its association with reversible cardiomyopathy. Given her underlying chronic anemia from SCD, however, a measured blood loss of 700 mL may represent a proportionally greater hemodynamic insult than the same volume in a patient without reduced baseline reserve, potentially masking clinically significant hypoperfusion despite blood loss below conventional thresholds. Sheehan syndrome is classically associated with significant postpartum blood loss and may present anywhere from days to years following delivery [1]. While acute Sheehan syndrome is rare, a recent case series identified 33 reported cases (including the index case) in the literature between 1990 and 2024 [4], in addition to two subsequently published case reports by Minamoto et al. and Dobiaš et al. [6,7]. A significant proportion of these cases lacked quantified blood loss, with classification often based on clinical evidence of hypovolemia rather than strict volumetric thresholds. The severity of pituitary dysfunction does not appear to consistently correlate with the documented degree of postpartum blood loss. Reports involving severe anemia, as well as the cases described by Kan et al. and Anfuso et al., in which early diabetes insipidus and acute-onset hyponatremia with severe anemia developed, respectively, suggest that clinically significant endocrine dysfunction may occur despite variable or incompletely documented degrees of postpartum blood loss [8,9]. These reports highlight that metabolic disturbances may be the earliest clinical indicators of pituitary injury in the acute postpartum period. Most reported cases present within hours to days postpartum and are associated with significant hemorrhage; however, acute presentations may be under-recognized, with many cases only diagnosed later when chronic hypopituitarism develops.

The onset of symptoms in our patient is consistent with the six-week window classically used to define acute Sheehan syndrome. She developed headache, dyspnea, dizziness, and palpitations on postpartum day 1, with a continuously evolving symptom course over the subsequent weeks, including the progressive development of fatigue, cold intolerance, dry skin, failure to lactate, and amenorrhea. This continuous, unremitting clinical course, without a symptom-free interval, is consistent with an early pituitary insult with progressive unmasking of multiple hormone deficiencies, rather than a new or delayed-onset process. However, biochemical confirmation of panhypopituitarism was not obtained until 11 weeks postpartum, as her presentation was repeatedly attributed to anemia and sickle cell-related hemolysis during multiple admissions, delaying the pituitary hormone testing that ultimately established the diagnosis. This case illustrates how the presence of a known chronic hemolytic condition such as SCD can anchor clinicians toward a more common explanation, delaying recognition of a rarer but clinically significant underlying diagnosis.

The association between SCD and acute Sheehan syndrome remains poorly characterized and underreported. One case report suggested that sickle cell trait alone may have acted as a potential predisposing factor for pituitary infarction in the absence of documented peripartum hypotension or major obstetric catastrophe [5]. In SCD, microvascular vaso-occlusion and severe hemolytic anemia during the peripartum period, together with physiological pituitary enlargement and increased metabolic demand, may theoretically increase susceptibility to pituitary ischemia and subsequent hypopituitarism even in the absence of massive hemorrhage or overt hypotension. However, a definitive epidemiological or causal association between SCD and Sheehan syndrome has not been established, and current evidence remains limited to isolated case reports and pathophysiological hypotheses. While clinical complexity may delay recognition, a high index of suspicion is required in postpartum patients with recurrent admissions or unexplained metabolic abnormalities. The absence of classic radiological features of Sheehan syndrome in the early postpartum period does not exclude the diagnosis, as characteristic findings such as pituitary atrophy and empty sella may evolve over time, as seen in our patient's serial imaging. In such cases, biochemical assessment remains central to establishing the diagnosis, particularly when early imaging is nondiagnostic. A limitation of this case is the absence of serial imaging between the initial presentation and the one-year follow-up MRI, precluding direct visualization of the trajectory of pituitary involution. As such, while the imaging findings are consistent with Sheehan syndrome, they cannot definitively distinguish this from a more generalized progressive pituitary injury of uncertain etiology evolving over the same period. We have therefore framed the diagnosis in this report as presumptive rather than confirmed.

It is unclear whether the cardiac dysfunction in our case was attributable to PPCM versus secondary cardiac dysfunction due to hypopituitarism in the context of acute Sheehan syndrome. Although cardiomyopathy associated with hypopituitarism is well described in the literature, only a small number of cases reporting the concomitant presence of Sheehan syndrome and cardiomyopathy have been documented [10-14]. There is a well-established association between hypopituitarism and cardiac dysfunction due to deficiencies in thyroid hormone, cortisol, estrogen, and growth hormone, which contribute to impaired myocardial contractility and reduced cardiac output [15,16]. Cortisol deficiency may contribute to acute cardiovascular compromise through reduced vascular tone and impaired myocardial responsiveness to catecholamines. Improvement in cardiac function following endocrine replacement therapy supports a potentially reversible contribution from hypopituitarism [10,11,14]. Distinguishing PPCM from cardiac dysfunction secondary to hypopituitarism in this case is not possible with the available data. The timing of onset, at 11 weeks postpartum, falls within the accepted window for PPCM (up to 5 months postpartum), and therefore does not argue against this diagnosis. Furthermore, because endocrine replacement therapy and guideline-directed heart failure treatment were initiated concurrently, the observed recovery of cardiac function cannot be attributed specifically to either intervention; it is equally consistent with reversal of hypopituitarism-related cardiac dysfunction or the natural course of resolving PPCM, which itself frequently improves with standard heart failure therapy alone. The co-occurrence of cardiac dysfunction with multiple other pituitary hormone deficiencies developing in parallel makes a unifying hypopituitarism-related etiology plausible, but does not exclude a concomitant or coincidental PPCM.

## Conclusions

Sheehan syndrome should be considered in any postpartum woman presenting days to years after delivery with unexplained hyponatremia, hypoglycemia, failure to lactate, amenorrhea, or other features of pituitary hormone deficiency, even in the absence of massive PPH or classic radiological features of Sheehan syndrome. This is particularly relevant in patients with SCD, where anemia, hemolysis, and recurrent transfusion requirements may complicate recognition of the underlying diagnosis. Early recognition and treatment are essential to prevent serious complications. Cardiac dysfunction associated with hypopituitarism may be reversible with appropriate hormone replacement and should be considered a potentially reversible cause of heart failure in patients presenting with severe pituitary hormone deficiencies.
